# Low-cost gel-filled microwell array device for screening marine microbial consortium

**DOI:** 10.3389/fmicb.2022.1031439

**Published:** 2022-12-16

**Authors:** Clelia Duran, Shiyi Zhang, Chongyang Yang, Maria Lorena Falco, Cristiana Cravo-Laureau, Chiho Suzuki-Minakuchi, Hideaki Nojiri, Robert Duran, Fumihiro Sassa

**Affiliations:** ^1^Universite de Pau et des Pays de l'Adour, E2S UPPA, CNRS, IPREM, Pau, France; ^2^Graduate School of Information Science and Electrical Engineering, Kyushu University, Fukuoka, Japan; ^3^Agro-Biotechnology Research Center, Graduate School of Agricultural and Life Sciences, The University of Tokyo, Tokyo, Japan; ^4^Collaborative Research Institute for Innovative Microbiology, The University of Tokyo, Tokyo, Japan

**Keywords:** gel-filled microwell array, bacterial screening, high-throughput, microbial consortium, microbial isolation method

## Abstract

In order to exploit the microbes present in the environment for their beneficial resources, effective selection and isolation of microbes from environmental samples is essential. In this study, we fabricated a gel-filled microwell array device using resin for microbial culture. The device has an integrated sealing mechanism that enables high-density isolation based on the culture of microorganisms; the device is easily manageable, facilitating observation using bright-field microscopy. This low-cost device made from polymethyl methacrylate (PMMA)/polyethylene terephthalate (PET) has 900 microwells (600 μm × 600 μm × 700 μm) filled with a microbial culture gel medium in glass slide-sized plates. It also has grooves for maintaining the moisture content in the micro-gel. The partition wall between the wells has a highly hydrophobic coating to inhibit microbial migration to neighboring wells and to prevent exchange of liquid substances. After being hermetically sealed, the device can maintain moisture in the agarose gels for 7 days. In the bacterial culture experiment using this device, environmental bacteria were isolated and cultured in individual wells after 3 days. Moreover, the isolated bacteria were then picked up from wells and re-cultured. This device is effective for the first screening of microorganisms from marine environmental samples.

## Introduction

To exploit the numerous beneficial microbes present in our environment, effective and rapid selection of beneficial microorganisms with useful functions from the vast number of microbes inhabiting our planet is essential; more than 5 × 10^30^ prokaryotic cells (Wooley et al., 2010) and more than 1 trillion (10^12^) microbial species (Locey and Lennon, 2016) are estimated to exist on the earth. The utilization of microorganisms as a natural resource has gained attention in many fields, including human health, agro-industry, environmental remediation processes, and chemical production using biotechnology. Moreover, microbes have been successfully utilized for the development of various products and industrial processes, such as the development of new anti-HIV (Banyal et al., 2021) and anti-cancer drugs, bioethanol production using highly functional yeast (Mohd Azhar et al., 2017), and establishment of wastewater treatment plants using microbial consortia (Cydzik-Kwiatkowska and Zielinska, 2016). Thus, microbial resource-based processes have become the key technologies in this century; they are particularly important in the fields of clean energy, biotechnology, drug discovery, and environmental technology.

The conventional method for screening microorganisms involves a microbial suspension extracted from an environmental sample (soil or any other natural sample), which is subjected to selection pressure on an agar plate containing adequate medium and screening components according to the criteria of microbial selection under controlled conditions, including constant temperature and oxygen levels. Then, pure microbial strains, collected from the colonies formed on the solid medium plate, are cultured for variable periods (from overnight to several days) in order to evaluate their metabolic functions. However, this approach has some disadvantages because it is time-consuming and requires several days, and the number of cultures that can be processed by hand is limited to several dozens. In addition, due to the competition among the different microorganisms present in an environmental sample, it is difficult to isolate microorganisms with low growth rate because of competitive inhibition by microorganisms with fast growth rate (Jiang et al., 2016).

In recent years, microfabrication techniques, such as Bio-Micro Electro Mechanical System (BioMEMS) and microfluidics have been used to develop microbial research tools (Weibel et al., 2007). Various devices have been developed for microbial culturing using microfluidic technology, such as a combination of optical tweezers (Enger et al., 2004), a tool for the evaluation of microbial motility by chemical gradient (Walker et al., 2005), and disease model devices by co-culture with human cells (Shah et al., 2016). Some devices can handle single-cell bacteria with sub-micrometer scale microfluidic structures (Balaban et al., 2004; Boedicker et al., 2009; Mannik et al., 2009; Moffitt et al., 2012; Taheri-Araghi et al., 2015). In particular, an advanced micro-liquid manipulation system using a poly dimethylpolysiloxane (PDMS) micro-pneumatic valve (Unger et al., 2000; Thorsen et al., 2002) has shown high versatility. It can be used for individual bacterial cell isolation and selective manipulation of solutions containing medium (nanoliter scale), as well as digital PCR (Ottesen et al., 2006) and microchemostat culture (Balagadde et al., 2005). However, it requires a special and expensive setup, including a large external drive pneumatic pump array system to drive each system.

On the other hand, high-throughput and low-cost microbial isolation and culture systems using droplets (liquid plugs) have been proposed (Boedicker et al., 2008; Walter et al., 2011; Guo et al., 2012; Jiang et al., 2016; Kaminski et al., 2016). However, it is difficult to track the growth of cells in individual droplets because the position of the droplet is indefinite. Therefore, statistical batch processing using a flow cytometer is widely used for analysis and bacteria isolation (Li et al., 2021). In addition, the aqueous phase (the droplet) and the oil phase surrounding the droplet are both liquids that allow the exchange of soluble substances between the droplets since it is difficult to completely block such exchange *via* the carrier fluid (oil phase), even if with the addition of appropriate surfactant to reduce the it (Courtois et al., 2009; Chowdhury et al., 2019).

A number of devices for culture evaluation in microenvironments using microfabrication techniques and microbial isolation culture array techniques, such as microbial cell patterns utilizing soft lithography, have been proposed (Cerf et al., 2008; Schirhagl et al., 2012; Arnfinnsdottir et al., 2015). In particular, devices that combine the color-coded droplet technique with microwell arrays enabled high throughput combinatorial co-culture analysis (Kehe et al., 2019). Moreover, large-scale bacterial culture techniques involving small gel spots of microbial suspensions formed on large gel plates (Srinivasan et al., 2013) and separation devices using micro through-hole array device placed on gel plates (Ingham et al., 2007) have been proposed. Since these devices can handle a large number of isolated microorganisms in independent compartments, individual growth can be tracked sequentially through microscopic observation, and all wells can be directly accessed for isolation. In recent years, agarose-made gel microwell arrays formed by soft lithography techniques that can be used for microorganism isolation (Zhang et al., 2019) and a technique for photo addressable recovery of isolates from wells based on photodegradable polyethylene glycol hydrogel membranes have been developed (Barua et al., 2020).

However, these devices are directly connected to a common gel plate at the bottom to prevent the drying of the gel throughout the duration of the culture, and it is difficult to prevent the exchange and diffusion of liquid substances *via* the plate as well as the droplet system. In a previous study, we developed a gel-filled microwell array device using photolithography that can be used for culture for more than 18 h without exchange or diffusion of liquid substances (Nomura et al., 2014). It contains a completely independent micro-gel medium with 3,900 wells per cm^2^. We used spacers and sealing technique for maintaining the small volume of the culture vessel, thus moisture could be prevented from entering the picoliter-scale gel. However, it was difficult to culture the cells for longer than one night with this device due to gel drying, which was a trade-off for the small size of the gel-filled wells. Moreover, the device was not cost-effective for one-time use due to the materials used for its fabrication, such as Pyrex glass, and photolithography processes. Furthermore, the sealing of the device required trained individuals.

In this study, we have developed a low cost, easy to use gel-filled microwell array device for long term microbial culturing. The device was fabricated using inexpensive plastic materials and fabrication methods.

## Materials and methods

### Bacterial strains and culture media

In order to determine whether bacteria can grow on the gel-filled microwell array device, we used *Pseudomonas putida* KT2440 (Bagdasarian et al., 1981) harboring the plasmid pBBR1MCS-5::mCherry, which possesses a red fluorescent protein (mCherry) encoding gene at the BamHI site of pBBR1MCS-5 (Kovach et al., 1995). We also used microorganisms from surface sediment marine samples (around the first 5 cm) collected at the Marina Port of Anglet (France) under a low tide. The sediments contained 31 ± 5 mg/kg of total petroleum hydrocarbon. An enrichment culture with acetate (4 g/l) as carbon source obtained from the hydrocarbon-contaminated marine sediments was used for the experiment.

The composition of the minimal salt medium (MSM) used in this study is as follows (g/L): KCl, 0.428; CaCl_2_.2H_2_O, 0.84; NH_4_Cl, 0.015; MgSO_4_.7H_2_O, 3.794; MgCl_2_.6H_2_O, 3.017; NaCl, 15.14; and Na_2_CO_3_, 0.15. After sterilization, the medium was supplemented with 100 μl of 50 mM phosphate buffer solution; 1 ml of trace element stock solution with the following composition (g/L): Fe (NH_4_)_2_(SO_4_)_2_.6H_2_O, 0.2; Na_2_SO_3_, 0.2; CoCl_2_.6H_2_O, 0.1; MnSO_4_.2H_2_O, 0.1; Na_2_MoO_4_.2H_2_O, 0.1; ZnSO_4_.7H_2_O, 0.1; AlCl_3_.6H_2_O, 0.04; NiCl_2_.6H_2_O, 0.025; H_3_BO_3_, 0.01; CuSO_4_.5H_2_O, 0.01; and 1 ml of V7 vitamin stock solution with the following composition (mg per 200 ml): vitamin B12, 10.0; p-aminobenzoic acid, 10.0; D-biotin, 2.0; acid nicotinic, 20.0; calcium pantothenate, 5.0; pyridoxine. HCl, 50.0; thiamine. HCl.2H_2_O, 10.0. Initial pH of the medium was adjusted to 7.0–7.2 with sterile 10 M NaOH. Filter sterilized trace element and V7 solutions were added to the sterile MSM as described in previous studies (Kovach et al., 1995; Alvarez-Barragan et al., 2021). The composition of the lysogeny broth (LB) is as follows (g/L): tryptone, 10; yeast extract, 5; NaCl, 10 (Sambrook, 2001). Gentamicin (Gm; 15 μg/ml) was added to the LB when needed.

### Structure of gel-filled microwell array device

**Figure 1:**
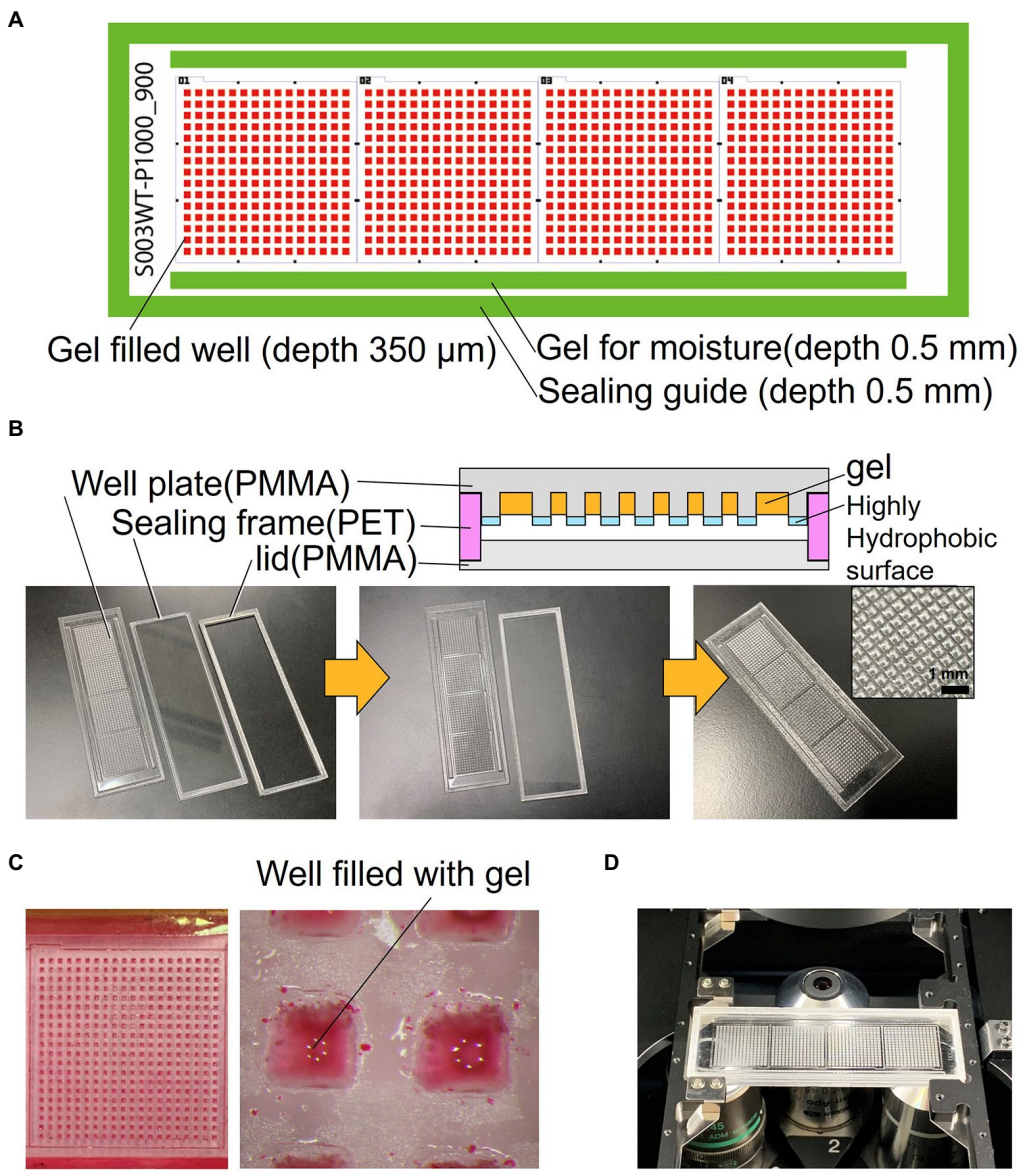
Structure of the resin-made gel-filled microwell array. **(A)** 2D arrangement of the device components. **(B)** Cross sectional view and photograph of the device. **(C)** Micrograph of gel-filled wells. **(D)** Photograph of the device mounted on microscope.

The schematics of the developed device are shown in Figure 1A. The device consists of a well-array plate, an airtight sealing lid made of polymethyl methacrylate (PMMA) for observation, and a sealing frame made of polyethylene terephthalate (PET) that serves as a spacer (Figure 1B). The footprint shape and size of the device are similar to that of a standard glass slide (76 × 26 mm); it can be mounted on a standard glass slide holder of a microscope. The thickness of the device is 3.7 mm after the three parts are stacked. The well-array plate is made of PMMA (thickness: 1.5 mm), and one plate has a total of 900 gel-filling wells, which are divided into four subareas with 15 × 15 wells each (size of a well: 600 μm × 600 μm; depth: 700 μm). Scales for visual observation are present around each well area. The top surface on the partition wall between the wells is coated with a highly hydrophobic material (contact angle of 110° or higher) to avoid cross-contamination caused by the transfer of bacteria between the wells.

In addition, rectangular gel-filling grooves (size: 65 mm × 1.5 mm; depth: 0.5 mm) are present on both sides of the four well areas. These grooves are filled with the same gel as that used for the medium to keep the micro-gel in the wells from drying on both sides of the device seal during incubation. The outer edge of the plate has a groove (1.8 mm) for sealing frame (0.5 mm depth), which keeps the spacing between the observation lid and the culture gel surface constant; it also maintains humidity inside the device. The sealing frame made from PET is rectangular in shape (width: 1.5 mm; height: 1.7 mm). This frame is designed to fit into the groove of the well-array plate described above. The lid for observation and the airtight seal are made of 1.5 mm thick PMMA and have a groove (0.5 mm) for fitting and assembling the sealing frame as well as the well-array plate. They can be easily stacked and disassembled. If necessary, the outer edge of the assembled device can be sealed with a removable adhesive (Paper Bond Ta-100, KOKUYO, Japan) or polyimide tape to increase airtightness.

### Fabrication of microwell array device

The device was fabricated using a CO_2_ laser engraving machine (LaserPRO C180II, GCC, Taiwan). The surfaces of PMMA (1.5 mm) and PET (1.7 mm) plates were cleaned with clean room grade wipes (BEMCOT PS-2, OZU CORPRATION, Japan). Then, they were set in a laser cutting machine, cut, and processed. Next, the device was brushed under running water to remove the dust formed during processing and then dried. The well-array plate was coated with a highly hydrophobic coating agent (FS-1060TH-2.0, Fluorotechnology, Japan) only on the top surface of the device to make the partitions between the wells hydrophobic; then the device was dried and thermally treated at 100°C for curing. Finally, to remove water-soluble impurities, the well-array plate was immersed in pure water overnight, and then dried. After assembly, the three completed parts were wrapped in aluminum foil and stored in a cool and dark place.

### Filling gel to microwell array device

After filling the gel medium into the wells, the fabricated device was used for culturing microorganisms. In the experiments described in this paper, the gel-filling process was performed before the inoculation step. Initially, the gel to be filled into the wells was melted in an autoclave. Next, the gel was poured into a glass Petri dish and kept warm at 60°C on a hot plate, and the stored device was disassembled and the well-forming plate was removed. The plate was immersed in pure water in a beaker at room temperature, the beaker was placed in a vacuum desiccator, and the pressure was reduced to approximately 1 kPa using a vacuum pump; air bubbles in the wells expanded due to the reduced pressure and were released from the wells. Consequently, the pressure returned to normal. Next, the wells of the plate were filled with pure water and immersed in the molten medium gel. To replace pure water with molten gel, the setup was not disturbed for 3 min. The Petri dish with the plate was placed in the vacuum desiccator again, and the pressure was reduced to remove the air bubbles present inside the well. The plate was then removed from the molten gel and cooled down for solidification in air. The remaining layer of the gel formed on the top surface of the plate was removed using polypropylene film and a PDMS spatula so that the water-repellent coating is exposed at the partitions between the wells. The plate was immersed in liquid medium, which had the same composition as that of the gel medium except for the gel component, at room temperature to prevent drying; the plate can be stored for several hours until the next step. Almost all of these operations were carried out on a clean bench.

### Inoculation and cultivation of bacteria

**Figure 2:**
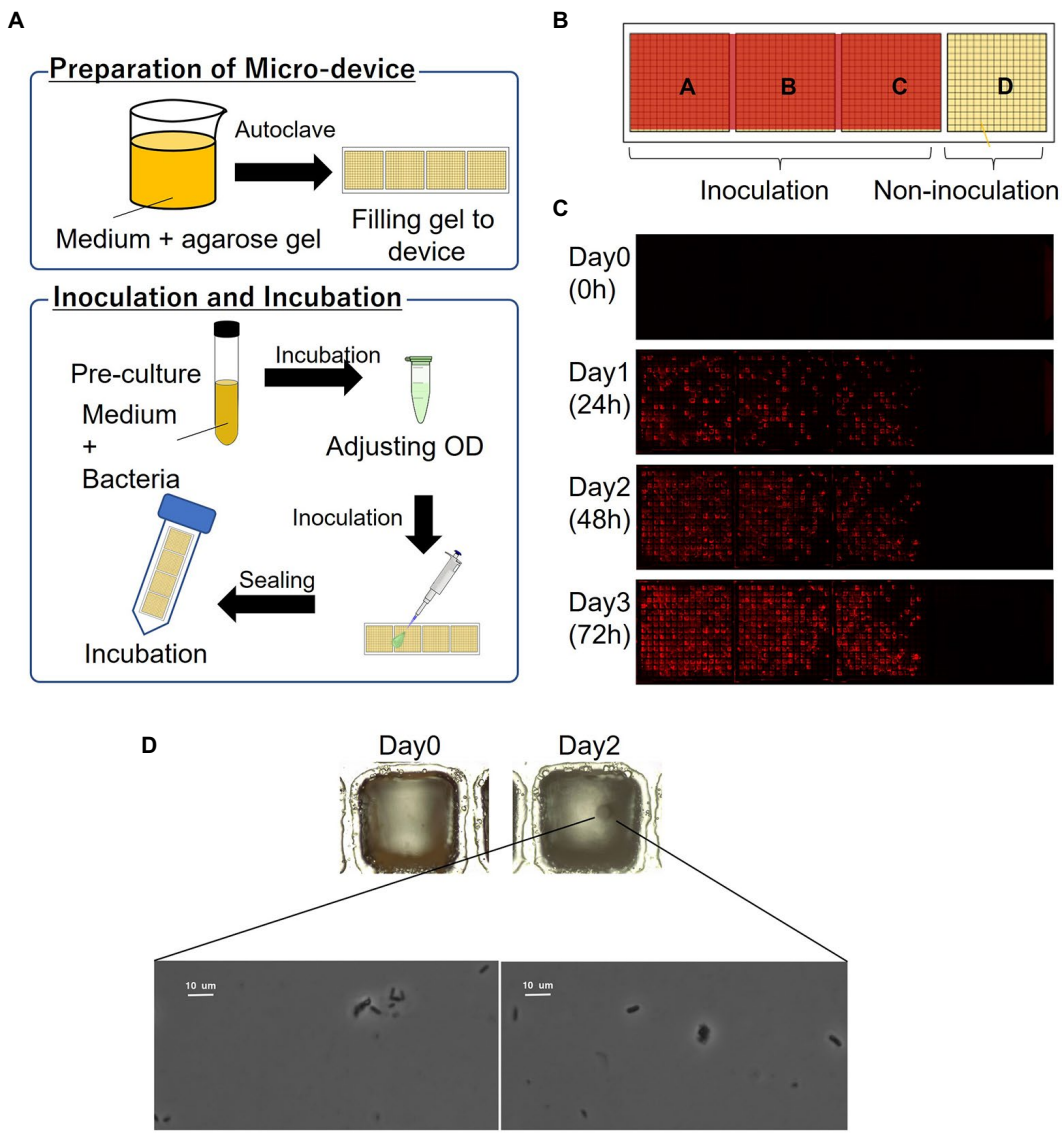
Culturing microorganisms in the gel-filled microwell array device. **(A)** Schematics of the culturing procedure using the device. **(B)** Arrangement of inoculation area on the device. **(C)** Micrographs of a gel-filled well just after inoculation to after 72 h of incubation. **(D)** Bacterial colony and bacterial cells from marine hydrocarbon-contaminated sediments obtained using gel-filled microwell array device. Microscopic observation of bacterial colonies (top; magnification ×100) and bacterial cells (down; magnification ×1,000).

The schematics of the inoculation and cultivation of bacteria are shown in Figure 2A. To cultivate *P*. *putida* KT2440 (pBBR1MCS-5::mCherry), LB containing Gm with 1% agarose was used to fill the wells of the device. The strain was pre-cultivated in LB supplemented with Gm at 30°C for 14–16 h. The optical density (OD) at 600 nm of the bacterial suspension was adjusted to 0.1 and diluted 10,000 times before inoculation. The resultant bacterial suspension was dispensed onto the gel-filled device by pipettes, and then removed. The partitions between the wells were hydrophobic so that the liquid remains as droplets only on the hydrophilic gel surface in each well. To remove the suspension liquid protruding from the few wells, the wells were air-blown and then sealed. The device was placed in a 50-mL conical tube with a wet paper towel and incubated at 30°C. mCherry-expressing cells were visualized by fluorescence microscopy.

To transfer the cells from the device into the liquid media, the wells on the device where mCherry-expressing cells existed were stabbed by a needle. The cells were inoculated into 96-deep well plates containing 1 ml of LB supplemented with Gm in each well. The plates were incubated at 30°C with shaking for 40 h. The growth in each well was evaluated by OD at 600 nm.

For the microorganisms from the marine samples, MSM (Dias et al., 2008; Alvarez-Barragan et al., 2021) containing 4 g/l sodium acetate as carbon source with 1% agarose was used to fill the wells of the device. The microbial suspension was adjusted to OD 0.1 at 560 nm and diluted 10,000 times before inoculation. The inoculation was performed as described above, and the device was incubated at 30°C.

## Results and discussion

**Figure 3:**
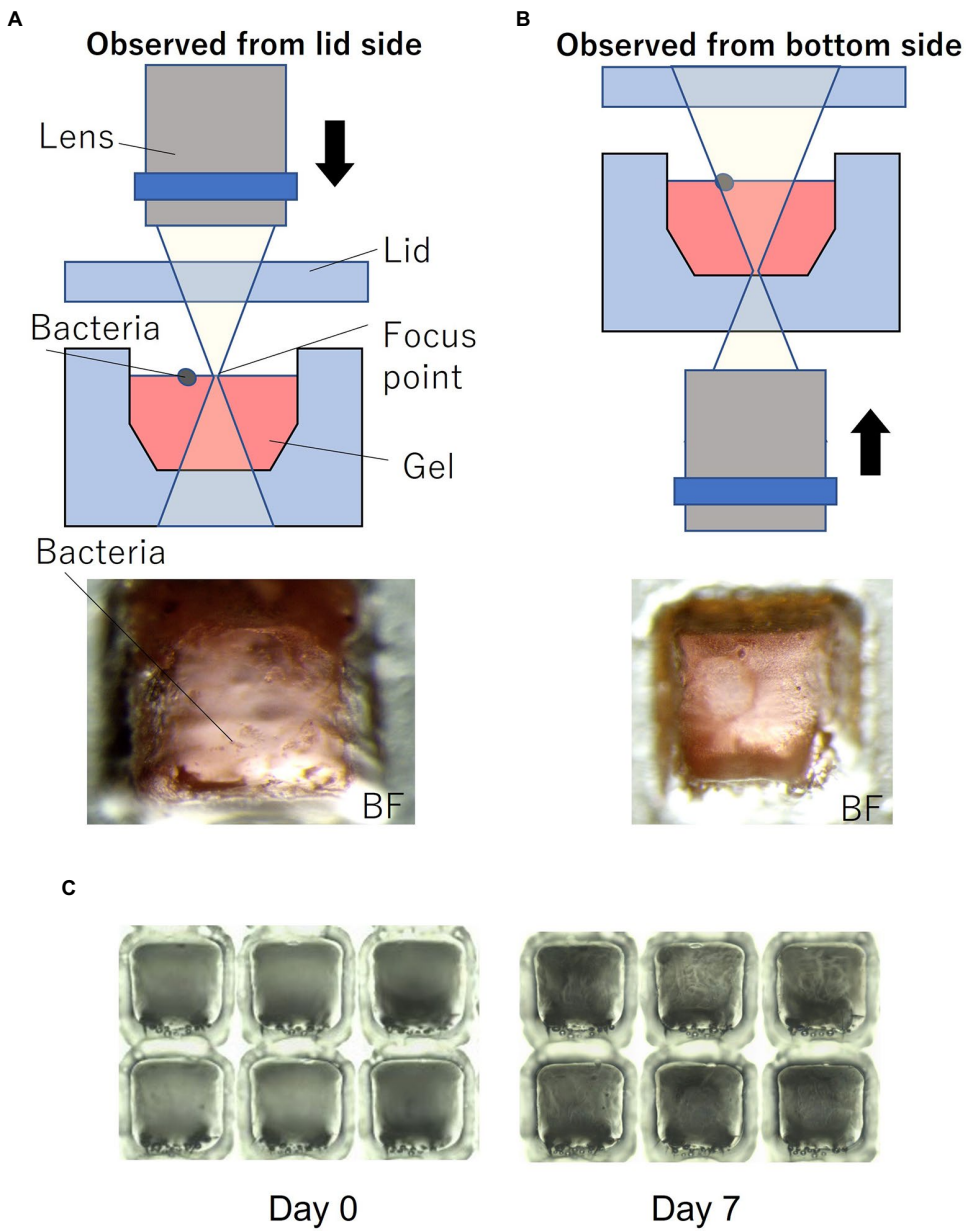
Microorganisms on the gel-filled microwell array. **(A)** Observation set up and micrograph from lid side. **(B)** Observation set up and micrograph from bottom side. **(C)** Micrograph of changes in the gel-filled device after 7 days at 30°C.

Figure 1C shows the device filled with gel using the method described in Section 2. To enable easy observation, the gel (1% agarose) used for the experiments was mixed with red food dye. Figure 1D shows the device sealed with a lid and mounted on a microscope. Most of the wells were filled with gel, and no gel was left in the partitions between the wells. Microscopic images of a typical gel-filled well from the lid side and the bottom side are shown in Figures 3A,B, respectively. The height of the gel was slightly lower than the height of the well. On this surface, microbial colonies can be optically observed even by bright-field microscopy.

To evaluate the moisture retaining property of this device, the device filled with gel was stored at 30°C. Figure 3C shows the changes in the gel-filled device after 7 days. After 7 days of storage, the volume of the gel decreased by 74% (*n* = 6), as observed by measuring the volume of the gel by changes in the distance from the focus point on the gel surface to the lid surface.

In order to evaluate the basic characteristics of the fabricated gel-filled device, a microbial culture experiment was conducted using *P*. *putida* KT2440 (pBBR1MCS-5::mCherry). The schematics of the procedure are shown in Figure 2A. By adjusting the concentration of the microbial suspension appropriately, each well was randomly inoculated with microbial cells with zero to several, often one. When a concentration of particle suspension is randomly inoculated into an array of a constant-size well, the number of bacteria entering each well is theoretically expected to follow a Poisson distribution (Hansen et al., 2016). In this experiment, the inoculated concentration was determined by a preliminary experiment in which the concentration of the suspension sample was adjusted. As shown in Figure 2B, a compartment of the device was not inoculated and acted as a control. A micrograph of gel-filled wells just after inoculation to after 72 h of incubation is shown in Figure 2C. The microbial colonies expressing mCherry were observed on the gel surface of the wells after incubating the device for 24 h. In addition, several wells without microbial growth (isolated wells) were observed, indicating that the movement of the microorganisms between the wells was inhibited. From the device incubated for 72 h, 46 wells were stabbed by a needle and the cells were transferred into a 96-deep well plate containing liquid medium. Bacterial growth was observed in 98% of the wells (Table 1), confirming that it is possible to pick up and re-cultivate the bacteria grown on this device.

**Table 1:** Growth of *P*. *putida* KT2440 (pBBR1MCS-5::mCherry) transferred from the gel-filled well-array device into a 96-deep well plate.

No.	OD at 600 nm
1	1.263
2	1.013
3	1.036
4	1.177
5	1.107
6	1.073
7	1.278
8	1.073
9	1.114
10	1.150
11	1.173
12	1.114
13	1.258
14	1.057
15	1.001
16	1.029
17	1.142
18	1.073
19	1.098
20	1.150
21	1.144
22	1.067
23	1.154
24	1.110
25	1.075
26	1.084
27	1.127
28	1.067
29	1.065
30	1.129
31	1.131
32	1.246
33	1.127
34	1.070
35	1.238
36	1.115
37	1.358
38	1.079
39	1.020
40	1.118
41	0.986
42	0.996
43	1.103
44	1.095
45	0
46	1.147

Then we applied this method to cultivate microorganisms from marine samples. An enrichment culture obtained from the hydrocarbon-contaminated marine sediments was used as the microbial suspension for inoculation. MSM (Dias et al., 2008; Alvarez-Barragan et al., 2021) containing acetate as carbon source with 1% agarose was filled on the device. A single bacterial colony was observed on the gel surface of a well after incubating the device for 2 days (Figure 2D). Then, the colony was picked and the bacterial cells were observed under a microscope (Figure 2D). Thus, the proposed culturing method using the new device allowed us to obtain isolated colony formed by viable bacterial cells, showing that our approach can be applied for high-throughput screening of cultivable microbial cells from stock marine microbial communities.

Enrichment culture is often used to select beneficial microorganisms from environmental samples. Many microorganisms with valuable functions, such as the degradation of xenobiotic compounds, have been obtained by enrichment culture. This method selects fast-growing microorganisms, which can be dominant in the specific competitive conditions used for the enrichment process. These selected microorganisms, however, are not always dominant when applied in the actual environment. To obtain various microbes, it is useful to avoid competition in the consortia. One possible method is spreading the microbial suspension before enrichment on the selective solidified media using Petri dishes, though it is time- and space-consuming. The gel-filled microwell array device developed herein showed a possibility of solving this problem. The 900 gel-filled wells on the glass slide-sized plate work as independent solidified media and can be easily used for the first screening of target microbes from environmental samples. We should note that this method is applicable only to microorganisms able to grow on gel-solidified media. Moreover, the compounds added to the media should be soluble in water; it is challenging to use highly hydrophobic compounds, such as polycyclic aromatic hydrocarbons, on this device as for any aqueous medium. Recently, Nakai and colleagues succeeded in the isolation of a phylogenetically novel *Rhodospirillales* bacterium using the prototype of the device used in this study (Nakai et al., 2021). This indicates that the device reported here has the potential to isolate a microorganism that has been overlooked by conventional screening methods.

## Conclusion

In this study, we fabricated a glass slide-sized gel-filled microwell array device from resin for microbial culture screening. The device was fabricated by laser processing of PMMA and PET, and it is a stand-alone container with a lid that can be hermetically sealed. The device has a total of 900 independent gel-filled wells, and the partitions between the wells are coated with a highly hydrophobic barrier to prevent cross-contamination between the wells. The device consists of a small number of parts and is designed to be sealed in a few steps so that it can be easily handled by non-specialists. The gel-filled wells of the device showed the presence of gel even after storage for 7 days.

The microbial culture experiments using this device confirmed the formation of microbial colonies in the wells isolated to a single or a small number of bacteria. In addition, the colonies picked up from these wells could be regrown in liquid medium. This device was successfully applied for the cultivation of environmental bacteria able to grow on acetate as a carbon source, demonstrating that it can be easily used to perform high-throughput isolation and re-cultivation of microorganisms. This device has been fabricated using inexpensive materials and is low-cost. In addition, the well size and depth can be easily changed by design according to the microbial sample and the culture period of each experiment. The device is appropriate as a first screening tool in the search for useful microorganisms under various culture conditions. It represents a general microbial research tool for the screening of microorganisms from environmental samples as well as the selection of clones from metagenomic libraries.

## Data availability statement

The raw data supporting the conclusions of this article will be made available by the authors, without undue reservation.

## Author contributions

RD and FS designed the experiments. CD, MF, and RD provided the new bacterial sample. CD, MF, CC-L, and CY performed bacterial experiments with the micro device. SZ and FS designed and fabricated the micro device. CD, MF, CS-M, CC-L, HN, RD, and FS analyzed data. CD, CS-M, HN, RD, and FS wrote the main text. All authors contributed to the article and approved the submitted version.

## Funding

This work was the financially supported by the JSPS KAKENHI (grant numbers: 19H05679, 19H05680, 19H05686, 21K14768, and 22H01502), Adaptable and Seamless Technology Transfer Program through Target-driven R&D (A-STEP) from Japan Science and Technology Agency (JST) (grant number: JPMJTM20GW), Initiative for Realizing Diversity in the Research Environment, MEXT Initiative for Realizing Diversity in the Research Environment, MEXT Initiative for Realizing Diversity in the Research Environment, and Japan-France Integrated action Program (SAKURA) (grant number: JPJSBP120213205, Japanese-French HYBAM project through the bilateral program PHC SAKURA, project no. 46981TD) and EU-Marie Skłodowska-Curie grant No. 892764.

## Conflict of interest

The authors declare that the research was conducted in the absence of any commercial or financial relationships that could be construed as a potential conflict of interest.

## Publisher’s note

All claims expressed in this article are solely those of the authors and do not necessarily represent those of their affiliated organizations, or those of the publisher, the editors and the reviewers. Any product that may be evaluated in this article, or claim that may be made by its manufacturer, is not guaranteed or endorsed by the publisher.

## Abbreviations

BioMEMS: Bio-micro electro mechanical system
LB: Lysogeny broth
MSM: Minimal salt medium
OD: Optical density
